# Quantitative SPECT: the time is now

**DOI:** 10.1186/s40658-019-0241-3

**Published:** 2019-03-04

**Authors:** John Dickson, James Ross, Stefan Vöö

**Affiliations:** Institute of Nuclear Medicine, University College London Hospital, Euston Road 235, London, NW1 2BU UK

**Keywords:** Quantification, SPECT, SPECT-CT, SUV

## Abstract

**Background:**

Quantification is one of the key benefits of nuclear medicine imaging. Recently, driven by the demand for post radionuclide therapy imaging, quantitative SPECT has moved from relative and semiquantitative measures to absolute quantification in terms of activity concentration, and yet further to normalised uptake using the standard uptake value (SUV). This expansion of quantitative SPECT has the potential to be a useful tool in the nuclear medicine armoury, but key factors must be addressed before it can meet its full potential.

**Discussion:**

Quantitative SPECT should address an unmet clinical need and give metrics that are clinically meaningful. Using the technique in a similar manner to PET with longitudinal assessments of disease in terms of SUV is one example that meets these criteria. Having metrics that are evaluated to ensure that they are correct, that are optimised to maximise their sensitivity, and that are transferrable to allow multi-centre learning and applicability to all users of the technology are other areas of quantitative SPECT that need to be addressed and that have specific challenges associated with them. Finally, ensuring quantitative SPECT is cost-effective in times when healthcare budgets are being squeezed is also very important.

**Conclusion:**

Quantitative SPECT offers the possibility to continue and expand the potential of quantitative nuclear medicine applications. The time is now to ensure that our community works together to make this potential a reality.

## Background

“Nuclear medicine imaging is an exquisitely sensitive method of assessing and quantifying physiological processes in vivo.” These three pillars of sensitivity, in vivo assessment of (patho)physiology, and quantification are the essence of the success of nuclear medicine imaging. Historically, nuclear medicine quantification has progressed from simple thyroid uptake measurements through to present day kinetic and standard uptake value (SUV) analysis using PET. More recently, single photon emission computed tomography (SPECT) quantification in terms of kBq/cc has become more common—predominantly because of the needs of radionuclide therapy. With this development, software manufacturers have taken on this use and made such quantification easier and more accurate and expanded it further to provide SPECT in terms of SUV. While SUV may have initially been a construct for F-18 fluorodeoxyglucose (FDG) positron emission tomography (PET) imaging [1], it is commonly used in non-FDG PET and is now being seen as an equally appropriate metric for SPECT. Compared to PET, fully quantitative SPECT in terms of SUV or kBq/cc offers a wider range of radiopharmaceuticals and applications, so the success of this technology should be straightforward. Yet uptake of the technology is slow. To understand the obstacles and potential possibilities of quantitative SPECT, we must consider what we require from a quantitative metric.

## Discussion

The recipe for success for imaging biomarkers has been previously discussed [2]. Based on these discussions, one can consider that for a quantitative metric to be successful it should:

- Address an unmet clinical need
- Be clinically meaningful
- Be evaluated
- Be optimised
- Be transferrable
- Be cost-effective

### Address an unmet clinical need

Imaging typically addresses clinical questions in two ways: as a single time point cross-sectional investigation to assist in patient diagnosis and staging or as a series multi-time point longitudinal studies to assess disease progression or response monitoring. For each of these scenarios, it is possible to identify use cases for quantitative SPECT.

**Fig. 1:**
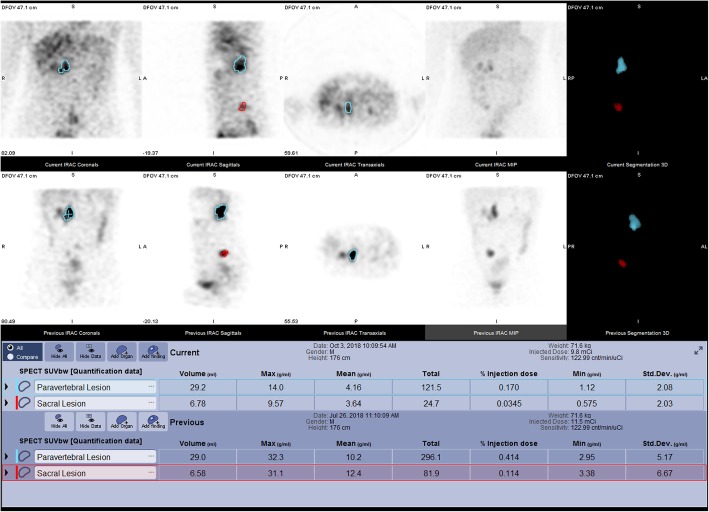
Iodine-123 mIBG imaging of a 19-year-old male patient with relapsed neuroblastoma. The upper imaging and result panel are from a post-mIBG therapy study, while the lower imaging and result panel are from pre-therapy imaging. The data from post therapy imaging shows a clear quantitative metabolic response in paravertebral and sacral lesions

An example of a longitudinal use case is in the field of theragnostics. Quantitative PET imaging is already used extensively in this field using Ga-68/Lu-177 peptides for diagnosis, staging, and treatment of somatostatin receptor tumours [3] and Ga-68/Lu-177 PSMA labels for the diagnosis, staging, and treatment of prostate cancer [4]. Similar pairings are also on offer with quantitative SPECT: I-123/I-131 MIBG for neuroendocrine tumours, and an alternative Tc-99 m/Lu-177 PSMA pairing for prostate cancer [5, 6]. For these use cases, quantitative SPECT can clearly be useful for assessing metabolic response in much the same way as their PET counterparts. This can be seen in Fig. 1 showing I-123 MIBG quantitative SPECT scans prior to and following therapeutic treatment with I-131 MIBG, where I-123-MIBG is the diagnostic agent. While visually it may be difficult to see a change post therapy, quantitative MIBG imaging in terms of SUV showed a metabolic response. Outside of theragnostics, longitudinal SPECT SUV assessment in musculoskeletal diseases is gaining interest and is being used in the clinical setting. For example, decrease in bone growth/osteoblastic activity in temporomandibular joint disorders or increase in bone activity in periprosthetic joint are suggested to be important in the clinical decision-making and surgery planning [7]. Other applications such as quantitative assessment of longitudinal presynaptic dopaminergic system, myocardial perfusion, sympathetic innervation, or amyloid deposition could also be envisaged. The challenges with longitudinal quantitative SPECT (as with longitudinal quantitative PET) are understanding the relevance of a change in the response metric. Further work will be required to understand the significance of changes before quantitative SPECT metrics are proven to be helpful in this arena.

**Fig. 2:**
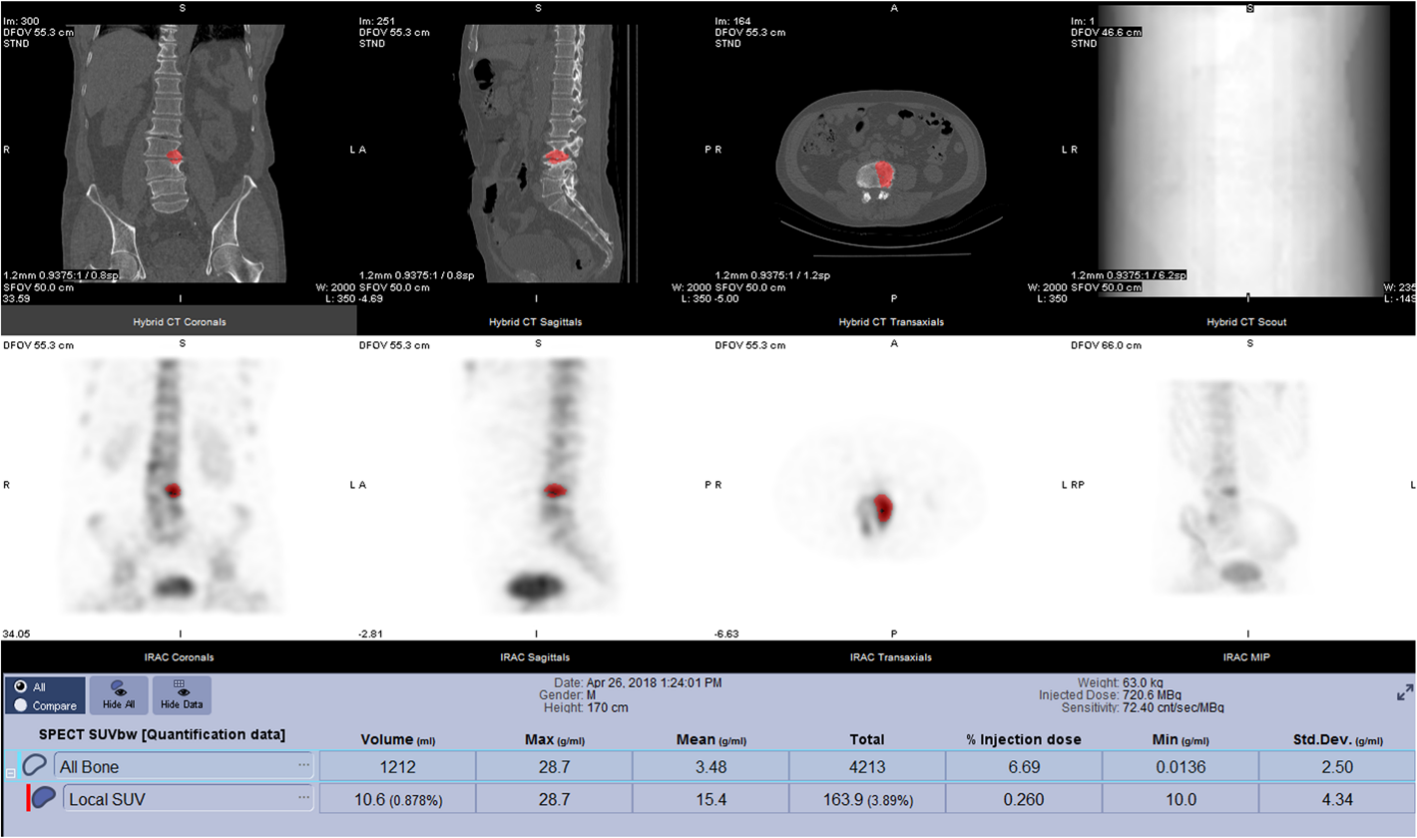
Quantitative bone SPECT of a 69-year-old female patient with a right hip prosthesis who is experiencing bone pain. Data show degenerative changes at the L3 and L4 endplates and elevated SUV

While longitudinal quantitative SPECT is a relatively easy target for quantitative SPECT, cross-sectional quantitative assessment in areas such as bone SPECT are also becoming of interest [8]. However, although it is relatively easy to determine a SPECT SUV value for a bone lesion (Fig. 2), the challenge is understanding the clinical relevance of the measured SUV. Whether a quantitative result is normal or abnormal requires some knowledge of what normal might be, which requires a normal quantitative range or database to compare to. In PET, the relevance of SUV values through an understanding of normal values was determined over many years and through many studies. The alternative of using a normal database is extremely challenging: because of the ethical, financial, and operational difficulties of collecting the data and also because, for example in the field of bone SPECT, it has been found that different components of the skeleton have very different normal values even before factoring in demographic information such as age, gender, weight, and height [9]. Visual assessment of data tends to rely on subjective evaluation assessment of contrast between normal and abnormal tissue, which may mean that uptake ratios rather than SUV or kBq/cc quantification may be adequate. Very little work has been done in this area, but with the advent of quantitative SPECT tools, it will be easier to explore this further.

Outside cross-sectional and longitudinal use cases for quantitative SPECT is the use case for dosimetry. The rapid expansion of theragnostics has led to an increasing demand on quantitative SPECT for dosimetry following nuclear medicine therapy. Assessing doses to organs at risk and target sites can help optimise the therapy by delivering the maximum dose to the target while keeping any damage to organs at risk to acceptable levels. The use of these strategies is being seen in prostate [10] and neuroendocrine [11] cancers, while pre-therapy dose estimates based on imaging are common with microsphere [12] therapies. While dosimetry is currently predominantly being used in research, following the introduction of new ionising radiation legislation (EURATOM 2013-59) which specifically calls for radiotherapeutic exposures to be “individually planned” and “appropriately verified” [13], there is an increasing appetite for performing dosimetry following routine administrations of therapeutic nuclear medicine. Up until recently, this was almost exclusively performed using manual processes but recent software developments by manufacturers have made this process significantly easier. While the usefulness of nuclear dosimetry to some is debatable [14] for those who rely on dosimetry, quantitative SPECT is an essential part of the process [15].

### Be clinically meaningful

Having quantitative SPECT values in terms of kBq/cc can be helpful for dosimetry but has little clinical relevance. As an alternative, standardised uptake value (SUV) is being proposed as a convenient method of quantifying uptake which also normalises to injected activity and body habitus. However, this and similar metrics such as SUV tissue ratios (SUVr) have assumptions associated with them related to the kinetics of the radiopharmaceutical being used, particularly related to the stability and non-reversibility of uptake. Fortunately, many radiopharmaceuticals such as MDP/HDP [16], HMPAO [17], and mIBG [18] have kinetic models that are the same or similar to the non-reversible two-tissue compartment model (2TCM) followed by FDG PET which makes the use of SUV and SUVr appropriate. Nevertheless, as with FDG, with time-varying SUVs and drug or food interactions a possibility, tighter imaging windows post injection and more stringent patient preparation may be required. Other radiopharmaceuticals have kinetic models that are not analogous to FDG and therefore may not be suitable candidates for SUV or SUVr use, i.e. Thallium-201 in myocardial perfusion imaging. For radiopharmaceuticals where the model is unknown, kinetic modelling using whole-body and SPECT imaging will be required to confirm that SUV and SUVr are appropriate metrics.

How to normalise for body habitus when using SUV is another area of investigation. Typically, normalisation is being made to body weight, but as with FDG, whether it is better to normalise to body weight or more complex body habitus measures such as lean body mass or body surface is still to be determined. Indeed, it could be argued that bone SUV assessments should be normalised to total bone activity given the almost exclusive accumulation in bone tissue. Other possible measurements in quantitative SPECT, such as the percentage of injected dose, also offer some usefulness, although mostly for dosimetry and research purposes.

### Be evaluated

As a relatively new technology, although some work has been performed in quantitative SPECT for dosimetry assessments, very little other than phantom work has been performed in this area to assess the accuracy and precision of measurements. Clearly, this is an area that needs further work, ideally through harmonised processes so that a supportive body of evidence can be gathered.

### Be optimised

This element of what makes a suitable metric is often overlooked. The precision and bias of imaging metrics can be determined statistically, but how good is good enough being mindful of biological and other uncontrollable factors that will be in our measurements. Optimisation of SPECT protocols can also be particularly challenging. In PET, where there have been successes in quantitative imaging, the same series of corrections are applied to all reconstructed data, with raw data almost exclusively reconstructed using iterative reconstruction techniques. However, in SPECT, iterative reconstruction is not always used, CT attenuation correction may not be available, and corrections for scatter are rudimentary compared to techniques established in PET. Efforts are being made to improve SPECT reconstruction and correction [19]. Nevertheless, the wide choice and availability of correction techniques in SPECT makes optimisation very challenging—particularly if metrics need to be comparable with data acquired at other centres. Fortunately, optimisation of other parameters for quantitative SPECT such as the acquisition parameters used is relatively easy to optimise.

### Be transferable

Transferability of metrics is something essential to the portability of patient results and will be very important for the community to understand the meaning of results at this early adoption stage of the technology. This is an area that has been addressed in PET with organisations such as EARL and QIBA and is now starting to be addressed with quantitative SPECT [20]. With this being a young technology, there are some initial differences in the implementation of quantitative SPECT systems which need to be understood. For example, there are differences in the calibration of these systems and also differences in the reconstruction methods and correction techniques being applied, while the use of third-party software may also produce different quantitative results from the same SPECT system. These differences could be a serious impediment to the success of quantitative SPECT and so need to be addressed.

### Be cost-effective

Given the recent introduction of this technology, the evidence of cost-effectiveness is yet to be proven. While quantitative SPECT for dosimetry may bring efficiency savings by reducing the numbers of unneeded therapies or dosing adjustments, work to prove the effectiveness of the technology for other applications will need to be done. The cost-effectiveness of longitudinal assessments of interventions seems relatively straightforward to assess through more studies and more patients; cross-sectional studies, however, are likely to be more challenging in the short term because of the need to understand the relevance of findings. It will only be through studies with biopsy, complementary data, and clinical follow-up where the relevance of cross-sectional findings will be validated. Finally, the cost of the technology will also need to take into account: CT for attenuation correction is essential and would require an upgrade from SPECT to SPECT/CT, while quantitative SPECT software currently has additional cost.

## Summary

Quantitative SPECT offers meaningful metrics which could provide clear benefits for longitudinal assessment of disease pre- and post-intervention and dosimetry assessments post radionuclide therapy, while cross-sectional use cases will require further assessment to determine clinical value. Work also needs to be done to prove the value of all these uses, and the cost-effectiveness of these applications also needs to be evaluated.

The key challenges lie in the optimisation and transferability of quantitative SPECT techniques. As a community, we need to maximise the sensitivity and specificity of our techniques and be consistent in the way we collect our data in order to gain the evidence to understand the clinical relevance of our findings be it in terms of defining normal ranges or the significance of any longitudinal change. There are also other challenges that need to be faced before this technology can be a success. Operational challenges must be overcome so that we can routinely measure pre- and post-injection activities to determine injected activity and so that we can measure patient heights and weights for normalisation to body habitus. Changes to the DICOM Standard are also necessary to allow quantitative SPECT metrics to be available to all through our PACS workstations and not be limited to professionals with access to nuclear medicine workstations.

With quantitative SPECT, we have a technology that is more accessible than PET and something that could be more powerful considering the plethora of SPECT applications available to us. It is our responsibility therefore to explore this technology and add it to the armoury of sensitive, quantitative, and physiological measurements that we can offer our patients.

## References

[1] Huang SC (2000). Anatomy of SUV. Standardized uptake value. Nucl Med Biol.

[2] O'Connor JPB, Aboagye EO, Adams JE, Aerts HJWL, Barrington SF, Beer AJ (2017). Imaging biomarker roadmap for cancer studies. Nat Rev Clin Oncol.

[3] Baum RP, Kulkarni HR, Carreras C (2012). Peptides and receptors in image-guided therapy: theranostics for neuroendocrine neoplasms. Semin Nucl Med.

[4] Afshar-Oromieh A, Avtzi E, Giesel FL, Holland-Letz T, Linhart HG, Eder M (2014). The diagnostic value of PET/CT imaging with the 68Ga-labelled PSMA ligand HBED-CC in the diagnosis of recurrent prostate cancer. Eur J Nucl Med Mol Imaging.

[5] EDELING C-J, FREDERIKSEN PBC, KAMPER J, JEPPESEN P (1987). Diagnosis and treatment of neuroblastoma using metaiodobenzylguanidine. Clin Nucl Med.

[6] Robu S, Schottelius M, Eiber M, Maurer T, Gschwend J, Schwaiger M (2017). Preclinical evaluation and first patient application of 99mTc-PSMA-I&S for SPECT imaging and radioguided surgery in prostate cancer. J Nucl Med.

[7] Suh MS, Lee WW, Kim Y-K, Yun P-Y, Kim SE (2016). Maximum standardized uptake value of 99mTc hydroxymethylene diphosphonate SPECT/CT for the evaluation of temporomandibular joint disorder. Radiology.

[8] Armstrong AJ, Anand A, Edenbrandt L, Bondesson E, Bjartell A, Widmark A (2018). Phase 3 assessment of the automated bone scan index as a prognostic imaging biomarker of overall survival in men with metastatic castration-resistant prostate cancer. JAMA Oncol.

[9] Cachovan M, Vija AH, Hornegger J, Kuwert T (2013). Quantification of 99mTc-DPD concentration in the lumbar spine with SPECT/CT. EJNMMI Res.

[10] Okamoto S, Thieme A, Allmann J, D’Alessandria C, Maurer T, Retz M (2017). Radiation dosimetry for 177Lu-PSMA I&T in metastatic castration-resistant prostate cancer: absorbed dose in normal organs and tumor lesions. J Nucl Med.

[11] Nicolas GP, Mansi R, McDougall L, Kaufmann J, Bouterfa H, Wild D, et al. Biodistribution, pharmacokinetics and dosimetry of 177Lu-, 90Y- and 111In-labeled somatostatin receptor antagonist OPS201 in comparison to the agonist 177Lu-DOTA-TATE: the mass effect. J Nucl Med. 2017. 10.2967/jnumed.117.191684. 10.2967/jnumed.117.191684 28450554

[12] Manceau V, Palard X, Rolland Y, Pracht M, Le Sourd S, Laffont S (2018). A MAA-based dosimetric study in patients with intrahepatic cholangiocarcinoma treated with a combination of chemotherapy and 90Y-loaded glass microsphere selective internal radiation therapy. Eur J Nucl Med Mol Imaging.

[13] European Council Directive 2013/50/Euratom of 5 December 2013 laying down basic safety standards for protection against the dangers arising from exposure to ionising radiation, and repealing Directives 89/618/Euratom, 90/641/Euratom, 96/29/Euratom, 97/43/Euratom and 2003/122/Euratom. 2014. p. 1–73.

[14] Deandreis D, Rubino C, Tala H, Leboulleux S, Terroir M, Baudin E (2017). Comparison of empiric versus whole-body/-blood clearance dosimetry-based approach to radioactive iodine treatment in patients with metastases from differentiated thyroid cancer. J Nucl Med.

[15] Ljungberg M, Celler A, Konijnenberg MW, Eckerman KF, Dewaraja YK, Sjogreen-Gleisner K (2016). MIRD pamphlet no. 26: joint EANM/MIRD guidelines for quantitative 177Lu SPECT applied for dosimetry of radiopharmaceutical therapy. J Nucl Med.

[16] Moore AEB, Blake GM, Fogelman I (2006). Validation of a blood-sampling method for the measurement of 99mTc-methylene diphosphonate skeletal plasma clearance. J Nucl Med.

[17] Pupi A, Castagnoli A, De Cristofaro MT, Bacciottini L, Petti AR (1994). Quantitative comparison between 99mTc-HMPAO and 99mTc-ECD: measurement of arterial input and brain retention. Eur J Nucl Med.

[18] Wu J, Lin S-F, Gallezot J-D, Chan C, Prasad R, Thorn SL (2016). Quantitative analysis of dynamic 123I-mIBG SPECT imaging data in healthy humans with a population-based metabolite correction method. J Nucl Med.

[19] Hippeläinen E, Tenhunen M, Mäenpää H, Sohlberg A (2016). Quantitative accuracy of 177Lu SPECT reconstruction using different compensation methods: phantom and patient studies. EJNMMI Res.

[20] Seibyl S, Dewaraja Y, Dickson J, Miyaoke RS, Obuchowski N, Frey E (2018). QIBA SPECT Biomarker Committee: overview and status update.

